# Psychometric Evaluation and Workflow Integration Study of a Tablet-Based Tool to Detect Mild Cognitive Impairment in Older Adults: Protocol for a Mixed Methods Study

**DOI:** 10.2196/25520

**Published:** 2021-05-21

**Authors:** Josephine McMurray, AnneMarie Levy, Paul Holyoke

**Affiliations:** 1 Wilfrid Laurier University Brantford, ON Canada; 2 SE Research Centre Markham, ON Canada

**Keywords:** cognitive dysfunction, dementia, neuropsychological tests, evaluation study, technology, aged, primary health care

## Abstract

**Background:**

With the rapid aging of the global population, experts anticipate a surge in the prevalence of mild cognitive impairment (MCI) and dementia worldwide. It is argued that developing more sensitive, easy to administer, and valid MCI screening tools for use in primary care settings may initiate timely clinical and personal care planning and treatment, enabling early access to programs and services. Including functional competence measures in screening tests makes them more ecologically valid and may help to identify cognitive deficits at an earlier stage.

**Objective:**

We aim to conduct a preliminary evaluative study comparing the sensitivity, specificity, and reliability of the BrainFx Screen (referred to as SCREEN hereafter), a novel digital tool designed to assess functional competence and detect early signs of cognitive impairment, with the Quick Mild Cognitive Impairment, a validated and highly sensitive tool that detects MCI in the older adult population. We will also investigate the perceived usefulness and integration of the SCREEN into primary care practice to identify demonstrable impacts on clinical workflow and health care providers’ (HCP) perceptions of its success as a screening tool. Patients’ perceptions of completing the SCREEN and its impact on their quality of life will also be explored.

**Methods:**

This study has a concurrent, mixed methods, prospective, and quasi-experimental design. Participants will be recruited from 5 primary care family health teams (FHTs; defined by multidisciplinary practice and capitated funding) across southwestern Ontario, Canada. Participants will include HCPs, patients, care partners, and FHT administrative executives. Patients 55 years and older with no history of diagnoses for MCI, dementia, or Alzheimer disease rostered in one of the FHTs participating in the study will be eligible to participate. Their care partners will help triangulate the qualitative data collected from patients. Participating FHTs will identify an occupational therapist from their site to participate in the study; this HCP will both administer the research protocol and participate in semistructured in-depth interviews and questionnaires. Principal component analysis will be conducted on the SCREEN data to understand the test components better. Tests comparing sensitivity, specificity, and test-retest reliability will assess the validity of SCREEN as a screening tool for MCI.

**Results:**

This paper describes the study protocol and its activities to date. Data collection was halted early because of COVID-19 restrictions on research activity, and data analysis is currently in progress.

**Conclusions:**

At the end of the project, we anticipate having an initial comparative evaluation of the SCREEN as a tool for early detection of MCI in primary care older adult patient populations. Resource constraints on this research study limit our ability to conduct a randomized controlled trial; however, the results will assist developers of the SCREEN in determining whether rigorous controlled testing is warranted.

**International Registered Report Identifier (IRRID):**

DERR1-10.2196/25520

## Introduction

### Background

More than cancer and cardiovascular disease, declining cognition threatens an individual’s ability to age in place by living independently at home alone or with family caregivers [1]. According to the Centers for Disease Control and Prevention, 1 in 8 adults (more than 12%) 60 years and older reported experiencing memory loss and confusion, and 35% of that group reported functional difficulties with tasks related to mobility and self-care that reflect basic activities of daily living (ADL) [2]. With the rapid aging of the global population [3], experts anticipate a worldwide surge in the prevalence of mild cognitive impairment (MCI) and dementia, and with it challenges to health care systems, the labor force, and the lives of those assuming caregiver roles [4,5]. Studies have reported a gap between expected and observed prevalence of MCI, partly because of concerns that screening might lead to loss of independence, such as the right to drive [6-8], and reliance on case finding as the primary method of evaluation [9]. However, earlier identification of individuals with MCI, where true dementia is not present but the reduced cognitive function is detectable, may provide health care professionals with an important window for intervention [10]. It is argued that developing more sensitive, easy to administer, and valid screening tools for MCI may initiate more timely clinical and personal planning and treatment, enabling early access to programs and services supporting aging in place rather than institutionalization [11].

### MCI Disorder

MCI is a neurocognitive disorder that describes a state between normal cognition and dementia is characterized by a slight but noticeable deterioration of cognitive abilities that predominantly impairs memory and thinking skills [12,13]. MCI is clinically distinct from dementia, which is marked by progressive and irreversible neurodegenerative changes leading to loss of functional competence and independence (ie, loss of both simple ADL and instrumental activities of daily living [IADL]—everyday activities that require intact higher-order complex cognitive skills to complete, including managing finances, preparing meals, driving, or administering medications) [14-17]. In general, individuals diagnosed with MCI retain both basic ADL and complex IADL; however, a subset of individuals present with observable and measurable impairments in some IADL related to cognitive decline that impacts their day-to-day function [18,19]. The notion of functional impairment in MCI remains controversial because it does not present consistently among those diagnosed with MCI [10,14,18], and there are no standard measurement tools or a clear operational definition of what functional impairment is within clinical and research communities [14,19,20]. To account for this ambiguity, contemporary diagnostic criteria acknowledge that individuals may present with minor impairments in functional IADL, whereas ADL are spared [14]. Depending on how cases are classified, the tests used, and the characteristics of the population, up to 42% of the world’s population older than 60 years have MCI, with an increasing prevalence among people older than 65 years [21]. In a general practice study, approximately 23% of MCI patients developed dementia within 3 years and three-quarters were stable or improved within the same period [22]. Although interactions between prescribed medications, alcohol or drug abuse, metabolic disorders, infections, and/or traumas may cause dementia-like symptoms not directly caused by dementia [23], only 0.6% of real dementias are reversible (0.29% partially and 0.31% fully [24]).

Early detection and diagnosis of declining cognition may not only allow health care providers (HCPs) to intervene in cases where the condition is reversible but also provide early and optimal management, tailored treatment planning, and timely access to education and psychosocial support to those at high risk for dementia [25-27]. However, screening for MCI in asymptomatic people 65 years and older is not recommended in Canada [9,28,29] or the United States [30,31]; case finding is the favored approach [32]. This is partly because of the limited availability of randomized controlled trials and clinical heterogeneity, which impedes our ability to generalize what may be small-to-moderate short-term improvements in cognitive function through early interventions such as exercise and cognitive training or pharmacotherapies [33-39]. Furthermore, dementia is a syndrome, not a disease [6,40,41], where the indicators are continuous and affected by a wide variety of factors such as education and genetics, and thus require expert clinical judgment for a definitive diagnosis [40,42]. Diagnosis is difficult because HCPs rely in part on patients to self-identify or informants to report symptoms of cognitive dysfunction [25]. In Canada, it is estimated that up to 10% of community populations aged 65 years and older have some form of undetected cognitive impairment [35], and in the United States, up to 76% of those who have experienced confusion or memory loss do not consult a health care professional [43]. Consequently, only 20%-50% of people with dementia are recognized and documented in primary care [44]. Asymptomatic screening is not recommended [45], and some believe that MCI screening may generally cause anxiety (although there is no supporting evidence [46]) and possible overtreatment of patients who are unlikely to develop dementia [22].

Not screening for cognitive changes may be a lost opportunity to identify individuals before they are biomarker-positive [47] and to improve the quality of their lives and those of their caregivers through timely planning. Patients report favoring access to information about their cognitive health as it provides a sense of personal agency on treatment planning and opportunities for shared decision making [48]. Other studies indicate that early diagnosis of cognitive impairment may lead to early interventions that improve patients’ and caregivers’ ability to cope [23]. Furthermore, a diagnosis of MCI or dementia is typically required to access support that may improve the lives of older adults with MCI and their caregivers. Hence, the ability to reliably diagnose early cognitive decline, including measures of functional impairment, may be an important gateway to receiving timely state-funded interventions. To this end, an attending clinician must not only confirm the presence of symptomatic changes in cognition but also that cognitive impairment is not caused by factors other than neurological decline, and then recommend appropriate interventions [10,37].

### Screening for MCI in Primary Care Settings

Typically, screening for MCI is triggered when someone raises concerns about their memory and thinking abilities with their general practitioner [49]. At present, no single screening or diagnostic tool has been identified as the gold standard for confirming the presence of MCI, largely because of its clinical heterogeneity [37,49-51]. Instead, MCI is clinically inferred based on a combination of the patients’ clinical history, subjective memory complaints, and objective measures of cognitive impairment on any number of validated cognitive tests along with complementary functional assessments, neuroimaging, and serology [13]. If MCI is suspected, additional neuropsychological assessments are necessary to rule out alternative explanations, including but not limited to dementia or delirium, and to aid in the process of determining the specific subtype of MCI present [10,25]. In Canada, patients receive a diagnosis of MCI, on average, 5 months after their initial memory complaint [49]. Confirming a diagnosis can also be a lengthy process, as general practitioners refer patients to geriatricians and neurologists for additional performance tests and often a combination of neuroimaging (computed tomography and magnetic resonance imaging) and bloodwork (thyroid and B12) [49].

The *current usual practice* screening tools for MCI used by clinicians in Canada are the Mini-Mental State Exam (MMSE) [52] and the Montreal Cognitive Assessment 8.1 (MoCA 8.1) [53]. Both are paper and pencil screens, administered in 10-15 minutes, scored out of 30, and validated as MCI screening tools across diverse clinical samples [53,54]. Universally, the MMSE is most often used, consisting of 20 items that measure orientation, immediate and delayed recall, attention and calculation, visual-spatial skills, verbal fluency, and writing. The MoCA 8.1, which was developed to improve the MMSE’s ability to detect early signs of MCI, places greater emphasis on evaluating executive function and language, memory, visual-spatial skills, abstraction, attention, concentration, and orientation across 30 items [53,55]. However, it was primarily designed to detect moderate-to-severe cognitive impairments and not the milder dysfunction characteristic of MCI, and it does not allow HCPs to determine the specific subtype of MCI [25,53,56,57]. The MMSE also lacks the high sensitivity needed to reliably detect subtle cognitive changes associated with MCI [57-61]. Moreover, the clinical efficacy of both screens for tracking changes in cognition over time is limited, as they are sensitive to practice effects with repeated administration [62].

Although not commonly used in Canada, the *Quick Mild Cognitive Impairment (Qmci)* screen is a more sensitive, specific, and validated screening tool for detecting MCI in older adults than other tests (including both the MMSE and MoCA 8.1) [59,63-67], and it is freely available for clinical or research use; instructional booklets and tear-off sheets are purchased separately. The Qmci evaluates 6 cognitive domains: orientation (10 points), registration (5 points), clock drawing (15 points), delayed recall (20 points), verbal fluency (20 points), and logical memory (30 points) [68]. The relative contribution of points from each subtest to the overall score complement findings that delayed recall, verbal fluency, and logical memory are the most accurate subtests for differentiating MCI from normal cognition [69]. It is not known whether Qmci is subject to practice effects. However, there is evidence to suggest that tests of logical memory are sensitive to practice effects among participants with both normal cognition and MCI [70]. Therefore, as the logical memory subtest on the Qmci makes the largest relative contribution to the overall screen score, it is possible that the Qmci may be subject to some degree of practice effects.

Designing cognitive screens with greater ecological validity (ie, designing questions that are reflective of relevant life activities [71]) to detect early changes in executive function may also improve early MCI detection [72], suggesting that including measures of functional competence on cognitive screens may be beneficial. Although measures of functional competence have been used to supplement cognitive or other neurological evaluations in the hope of improving diagnosis and outcomes, its value is not fully understood [71], particularly concerning how to assess functional competence in early-stage MCI [20]. Finally, aside from the need for MCI screening tools in the primary care setting to be psychometrically tested, they should also be easy to administer, accessible, efficient, and affordable [64,73].

BrainFx is a for-profit firm that creates proprietary neurological assessment software designed to identify signs of brain function impairment. The BrainFx Screen (SCREEN) is an unvalidated, digitally administered, 15-minute, 7-question screen designed to identify early signs of MCI by assessing functional deficits that may not be readily identified by existing screens (refer to Table 1 for a summary of SCREEN activities), such as the MoCA 8.1, MMSE, and Qmci. The SCREEN is a short version of the BrainFx 360 Performance Assessment, which is designed to assess cognitive, physical, and psychosocial areas of neurofunction [74]. This is a 90-minute test administered digitally to test 26 cognitive domains across 49 tasks that are timed, scored, and subsequently compared with the Living Brain Bank (LBB), a database of all BrainFx 360 and SCREEN tests collected to date. The 7 activities used on the SCREEN were taken directly from the BrainFx 360 on the basis of clustering and regression analyses of LBB records in 2016 (N=188) [75]. The reliability of the BrainFx 360 has been validated in healthy adults (mean 22.9 years of age, SD 2.4 years), and results suggest that the overall test-retest reliability of the tool is high (intraclass correlation coefficient=0.85 [74]); however, only 2 of the 7 cognitive domains selected for the SCREEN have reliability coefficients above 0.70 (visual-spatial and problem-solving abilities). To date, BrainFx 360 has been used in clinical settings to assess neurofunction among youth and in a variety of other rehabilitation settings.

**Table 1:** Summary of SCREEN activities.

Activity	Description	Time to complete (s)
Abstract reasoning	Twenty everyday items are displayed, and the patient touches the item on the screen and slides each item, one at a time, into 1 of the 5 categories into which they best belong, while being timed.	90
Constructive ability	Two rounds of a photo being displayed and breaking into 9 pieces. The patient touches the pieces and slides each piece into a grid to reassemble, while being timed.	90
Prioritizing	Five everyday activities or tasks are presented, and the patient is told what time of day it is (eg, 7 PM), and the patient touches the screen and slides each item to prioritize the order in which the activities or tasks should be completed.	60
Numerical problem solving	Ten math questions requiring 1- or 2-digit answers are presented for a patient response using a numerical pad (+, −, ×, and /) while being timed.	90
Visual-spatial ability	Two rounds of patient selecting (by touch) into which shape a word fits best, while being timed.	30
Divided attention	The patient watches a pot on the stove about to boil over (denoted by boiling water and red signal) and must touch the pot and move it to the sink to dump out the water, while also touching the screen to match as many objects as they can within the kitchen scene.	90
Route finding	A map is presented with roads and multiple locations. In the first round, the patient traces the most efficient route between 2 locations, while being timed. In the second round, the patient traces the most efficient route between 2 locations but is instructed to make 2 stops on the way, while being timed.	90

The objectives of this research study are as follows:

1. To evaluate the psychometric properties of the SCREEN to assess functional competence and detect early signs of cognitive impairment when administered in a primary care setting to adults 55 years and older
2. To investigate the integration and use of the SCREEN in primary care practice and any demonstrable impact on clinical workflow and planning
3. To explore HCPs’, patients’, and care partners’ perspectives on adopting and using the SCREEN.

## Methods

### Study Design and Setting

The study has a concurrent, mixed method, prospective, and quasi-experimental design. Participants will be recruited from 5 primary care family health teams (FHTs; defined by multidisciplinary practice and capitated funding) across southwestern Ontario, Canada. FHTs that employ a registered occupational therapist as staff will be eligible to participate in the study, and participating FHTs will receive a nominal compensatory payment for their time spent collecting data for the study by administering the SCREEN, Qmci, and Geriatric Anxiety Scale (GAS) [76] screening tools; training; and communicating with the research team. A multipronged recruitment approach will be used in this study. All participants (HCPs, patients, care partners, and FHT administrative executives) will be assigned a study identification number that allows for category but not individual classification.

### Study Participants and Recruitment

#### Patients

Patients 55 years and older with no history of diagnoses for MCI, dementia, or Alzheimer disease rostered in one of the FHTs participating in the study will be eligible to participate. The age of eligibility includes those 55 years and older to capture an at-risk population with no current diagnosis of MCI who might be healthy or experiencing early symptoms of MCI that may or may not be apparent to the participant. Prospective participants may also be excluded based on a diagnosis with any of the following conditions that are associated with MCI or dementia-like symptoms (Textbox 1): major depression requiring hospitalization, psychiatric disorders (ie, schizophrenia and bipolar disorder), psychopathologies, epilepsy, substance use disorders, and sleep apnea (without the use of a continuous positive airway pressure machine [77]). These criteria have been used in similar MCI screen validation studies to exclude participants that may confound the interpretation of the screen results because they are experiencing symptoms of MCI or dementia, not because of the true presence of MCI but rather unrelated conditions. The use of tablets to complete the SCREEN requires that participants are able to read and think in English, discern color, and read 12-point font on the tablet and that they have adequate hearing and vision to interact with the administering HCP and adequate hand and arm function to manipulate and hold the tablet. Exclusion criteria, therefore, include colorblindness or any disability that impairs a person’s ability to hold and interact digitally with the tablet. Finally, patients must be available to participate in a minimum of 2 screenings, performed 3 months apart, and an entry and exit interview to participate in the study. Prospective participants will be required to be rostered with 1 of the 5 participating FHTs to ensure that HCPs can access their electronic medical record (EMR) and that there was a physician responsible for follow-up referral. Before study enrollment, HCPs will be required to screen their EMR to verify participant eligibility and, on an ongoing basis, update their EMR with the results of the MCI screens.

**Eligibility criteria for patient participants.**

**Inclusion criteria**

- Aged 55 years and older
- Able to read and think in English
- Rostered with a participating family health team
- Depression:
- Symptom-free for the last 6 months
- No history of hospitalization
- Use of low-dose antidepressants
- Brain injury
- Stroke
- Taking prescription neuroleptics, hypnotics, or antiepileptics medications:
- Whether taken for pain and not epilepsy
- Low dose
- Stable (no dose or medication change for the last 6 months)

**Exclusion criteria**

- Less than grade 6 education
- Diagnosis of mild cognitive impairment, dementia, or Alzheimer disease
- Color blindness
- Paralysis (in hands)
- Physical handicap that may influence test results
- Epilepsy
- Severe vision or hearing impairments (hearing aids are acceptable)
- Psychopathology
- Major depression (that has required hospitalization)
- Diagnosed psychiatric disorders
- Diagnosis of condition with susceptibility to causing dementia or cognitive deficits
- Alcohol or drug dependence
- Sleep apnea (with no use of continuous positive airway pressure machine)

Recruitment of patients will include diverse media strategies in both clinical and community settings. At the FHT, recruitment posters and 1-page summaries of the study will be posted in waiting rooms; exam rooms; and the FHT’s website and social media platforms, including Facebook and Twitter, where available. Recruitment posters will also be posted at public establishments local to the FHT (eg, YMCAs, libraries, pharmacies, and recreational sites). Interested participants may self-identify to the FHT HCP via telephone or to the research team via a dedicated study email or phone number. The research team will host information sessions at each participating FHT to provide clinical and nonclinical health team staff with information about the study to support seamless recruitment and onboarding of new patient participants.

#### Care Partners

Once enrolled in the study, the HCP will ask patients to identify a care partner (defined as someone who might be concerned about and or interested in the patient’s well-being), if there is one, who might be interested in participating in qualitative interviews and questionnaires as part of the study. This is not a requirement of the study; qualitative data collected from care partners will help triangulate the data collected from the patient participants. There are no eligibility requirements for care partners other than having a self-identified relationship with the patient and being able to read and write in English.

#### Health Care Providers

Participating FHTs will identify 1 occupational therapist from their site to participate in the study. This HCP will both administer the research protocol and participate in semistructured, in-depth interviews and questionnaires. To be eligible to participate, the HCP must have the approval to participate from the appropriate corporate agent (the Executive Director of the FHT). Before starting data collection, the HCP must complete a web-based training program—consisting of 3 self-directed training modules—and learn how to administer the Qmci to become a certified BrainFx administrator. The research team will conduct in-person training to cover the research protocol and administrative processes.

#### FHT Administrative Executives

Where available, the managing director or equivalent at the FHT will be interviewed to better understand contextual factors such as workload, funding, and patient population characteristics that may impact the results.

### Ethics and Consent

The study protocol has been reviewed and has received ethics clearance from the Wilfrid Laurier University Research Ethics Committee (ORE# 5820) and has been reviewed and approved through each FHT’s research approval process. All participants (HCPs, patients, care partners, and administrative executives) will read and sign an information and informed consent package before participating in the study. We will conform to recommendations for acquiring informed consent and conducting qualitative interviews with persons with dementia when recruiting patients who may be affected by a neurocognitive disease [78-80]. During oral informed consent, we will use plain language, repeat information that is not understood, and ask the participant to explain their understanding of what the study entails. During the interview, we will use plain language; be prepared to repeat questions as needed and ask questions in a different way, possibly using the participant’s own words to rephrase; allow participants ample time to respond to each question; and provide cues to what they were saying if they lose train of thought. If they dwell overly long on a particular question, we will validate the meaningfulness of their response, gently redirect them to the next question, monitor for fatigue, and allow them to continue the interview at another time. Participants will be informed that they can choose not to answer the questions asked to them. All participants will be assigned a study participation number, and when reporting, identifying information will be removed from verbatim quotes if approval has been provided for use.

### Measures

#### The 10-Item GAS

The GAS-10 is a 10-item self-report screen for anxiety in older adults [81] developed for rapid screening of anxiety in clinical settings (GAS-10 is the short form of the full 30-item GAS [82]). The screen includes 10 questions taken directly from the GAS that measure somatic (ie*, I felt tired*), cognitive (ie*, I could not control my worry*), and affective (ie, *I was irritable*) symptoms of anxiety that reflect those used to diagnose anxiety disorder in the *Diagnostic and Statistical Manual of Mental Disorders*, fourth edition, text revision. Participants will be asked to use a 4-point Likert scale (0=not at all, 1=sometimes, 2=most of the time, and 3=all of the time) to rate how often they have experienced each symptom during the past week, including the day of the visit [82]. The GAS-10 has a maximum score of 30, with higher scores indicating higher levels of anxiety [81-83]. Although 3 subscales have been identified, the GAS-10 is reported to be a unidimensional scale of general anxiety [84,85]. Validation of the GAS-10 suggests that it is optimal for assessing average to moderate levels of anxiety in older adults, its total and subscale scores are highly and positively correlated with the GAS, and it possesses high internal consistency [81]. This tool will assess the patients’ anxiety level as it relates to screening for cognitive impairment at the time of the assessment and any change in subjective ratings after completion of the MCI screen and between visits. Although the association between neuropsychological assessment anxiety and test performance is unclear [86,87], the inclusion of a pre-post anxiety measure will allow researchers to control this issue and to explore any variation in performance or anxiety related to tablet technology. Given the exclusion criteria for this study (Table 1), we do not anticipate high levels of nontest anxiety.

#### The SCREEN

The SCREEN (version 0.5, beta) will be administered on a tablet (ASUS ZENPAD 10.1“ WXGA IPS Display, 1920×1200), powered by a quad-core 1.5 GHz, 64-bit MediaTek MTK 8163A processor with 2 GB RAM and 16 GB storage. The tablet comes with a tablet stand for optional use and a dedicated stylus that is recommended for completion of a subset of activities. At the start of the study, HCPs will be provided with identical tablets, preloaded with the BrainFx app software for use for the duration of the study.

Using a standardized administration protocol developed by BrainFx, the HCP will instruct the patient to use either their finger or the provided stylus to complete the SCREEN. Following acclimation to the tablet, the patient will be required to complete a short survey to collect demographic information (eg, age, the highest level of education attained) and any history of pre-existing conditions and questions about the patients’ state of well-being at the time of testing (eg, self-reported concerns about their thinking, mood, hours slept, and pain). The questionnaire will be immediately followed by 7 activities that are modeled after everyday real-world actions purported to evaluate functional competence related to a variety of cognitive domains, including abstract reasoning, divided attention, or visual-spatial abilities (refer to Table 1 for a detailed description of the activities). Tasks will be timed and digitally scored, and an activity score for each activity will be generated based on a combination of the patients’ accuracy (ie, number of correct responses) and processing speed (ie, speed of completion). The relative weight that accuracy and processing speed contribute to the activity score is proprietary to BrainFx and is the same for each of the 7 activities.

At the end of each SCREEN, the patient will be prompted by the app to consent to contribute their scores to a database of results maintained by BrainFx, known as the LBB. The mean and SD of the LBB database will be updated in response to the addition of every new SCREEN. The patient’s performance on the SCREEN will be evaluated by comparing their results with the global reference population (ie, all available SCREEN results in the LBB at the time of testing). Filters are available that allow the HCP to compare the patients’ results with subcohorts using factors such as gender, education, age, or primary diagnosis. Individual SCREEN results reports display the individual’s activity score, the LBB mean (for the global reference population unless the operator selects subcohorts based on selected filters), and whether the activity score falls within 1 SD of the LBB mean. If the patient’s activity score falls 1 or more SD below the global mean, it is classified as an *area of challenge*. The HCP is instructed to use their clinical judgment to interpret the results by applying any number and combination of filters relevant to the patient. For the purpose of this study, the patient’s performance will be compared against all results in the LBB completed by people aged 55 years and older at the time of testing.

#### The Qmci Screen

The Qmci is a sensitive and specific screen that differentiates normal cognition from MCI [63,65]. The HCP will administer the screen by asking the patient questions and recording their response on a dedicated Qmci assessment form provided by the screen developers. The patient will be required to answer 1 question via paper and pencil. The Qmci takes approximately 5 minutes to complete, is scored by hand out of 100 points, and evaluates 6 cognitive domains: orientation (10 points), registration (5 points), clock drawing (15 points), delayed recall (20 points), verbal fluency (20 points), and logical memory (30 points) [68]. The overall cut-off score to distinguish normal cognition from MCI on the Qmci is ≤67, from cognitive impairment (MCI or dementia) ≤62, and dementia alone ≤54 [88]. Although not as broadly adopted as the MoCA 8.1 in Canada, its psychometric properties, administration time, and availability for use suggest that Qmci is the optimal market assessment tool for MCI screening in FHT settings.

#### The Task Technology Fit Questionnaire

In the Task Technology Fit (TTF) model, technologies refer to any tool(s) used to complete a task, and the task itself is any number of actions performed to complete the task. Operationally, *fit* is defined as the extent to which the technology assists in completing these necessary actions [89,90]. Building on the TTF, Goodhue and Thompson [89] introduced the technology-to-performance chain model to acknowledge, first, that measuring perceived net benefit to use requires that the technology be used to complete the task for which it is designed and, second, to factor in the impact of related social norms or personal attributes on the evaluation of the technology and its utilization. Evidence suggests that the better the fit between the characteristics of the technology and task, the better the impact technology has on performance, which positively influences its utilization [89,90]. TTF models have been used to evaluate technologies across diverse sectors, including health care [91] and education [92], via questionnaires that tap constructs related to the technology, such as perceived satisfaction, reliability, the accuracy of the task, task completion time, ease of use or training, risk, and trust [90]. Across studies, individuals are asked to rate their perspective on the technology using a 7-point Likert scale ranging from entirely disagree (1) to entirely agree (7), and questions are tailored to address the technology and research questions at hand [89].

In this study, the questions on the TTF questionnaire are designed to measure how the SCREEN system (ie, assessment, handouts, hardware, and technical support) influences the HCP’s ability to screen for MCI. Tasks include, but are not limited to, collecting data from the patient that are relevant to MCI and using those data to make necessary decisions (eg, the decision to refer patients for further neuropsychiatric evaluation or provide particular intervention recommendations for MCI [37,51]). HCPs will be asked to rate the SCREEN system according to how the technology impacts the characteristics of their tasks and their ability to perform them, user satisfaction, utilization, and their perceived net benefits to using the tool [89,92].

#### Zarit Burden Interview

Care partners will complete the 12-item Zarit Burden Interview (ZBI) [93] as part of their entry or exit interviews scheduled within 1 month of their partner’s first and last screening appointments. The ZBI is the most common self-report screen used to measure subjective burden reported by people caring for those with chronic health conditions who require, over time, increasing support for managing their day-to-day ADL and IADL (ie, cognitive impairment) [94,95]. The 12-item ZBI [94] is one of several validated short-form screens used for brevity in place of the full 22-item ZBI [96]. The 12-item ZBI is reported to measure 2 dimensions of burden (personal and role strain) [94], is highly and positively correlated with scores on the full 22-item ZBI, presents with high internal consistency, and is a reliable tool for measuring changes in caregiver burden over time [93,94,97,98]. Participants are asked to use a 5-point Likert scale (0=never, 1=rarely, 2=sometimes, 3=quite frequently, and 4=nearly always) to rate how they feel in response to items such as, “Do you feel strained when you are around your relative?” and “Do you feel that your health has suffered because of your involvement with your relative?” [94]. Participants can score a maximum of 48 points, and scores equal to or higher than 17 are classified as a high or severe burden [94,99]. Data from this questionnaire will be used to triangulate the test results, impact, and patient self-reported anxiety.

### Data Collection and Procedures

**Figure 1:**
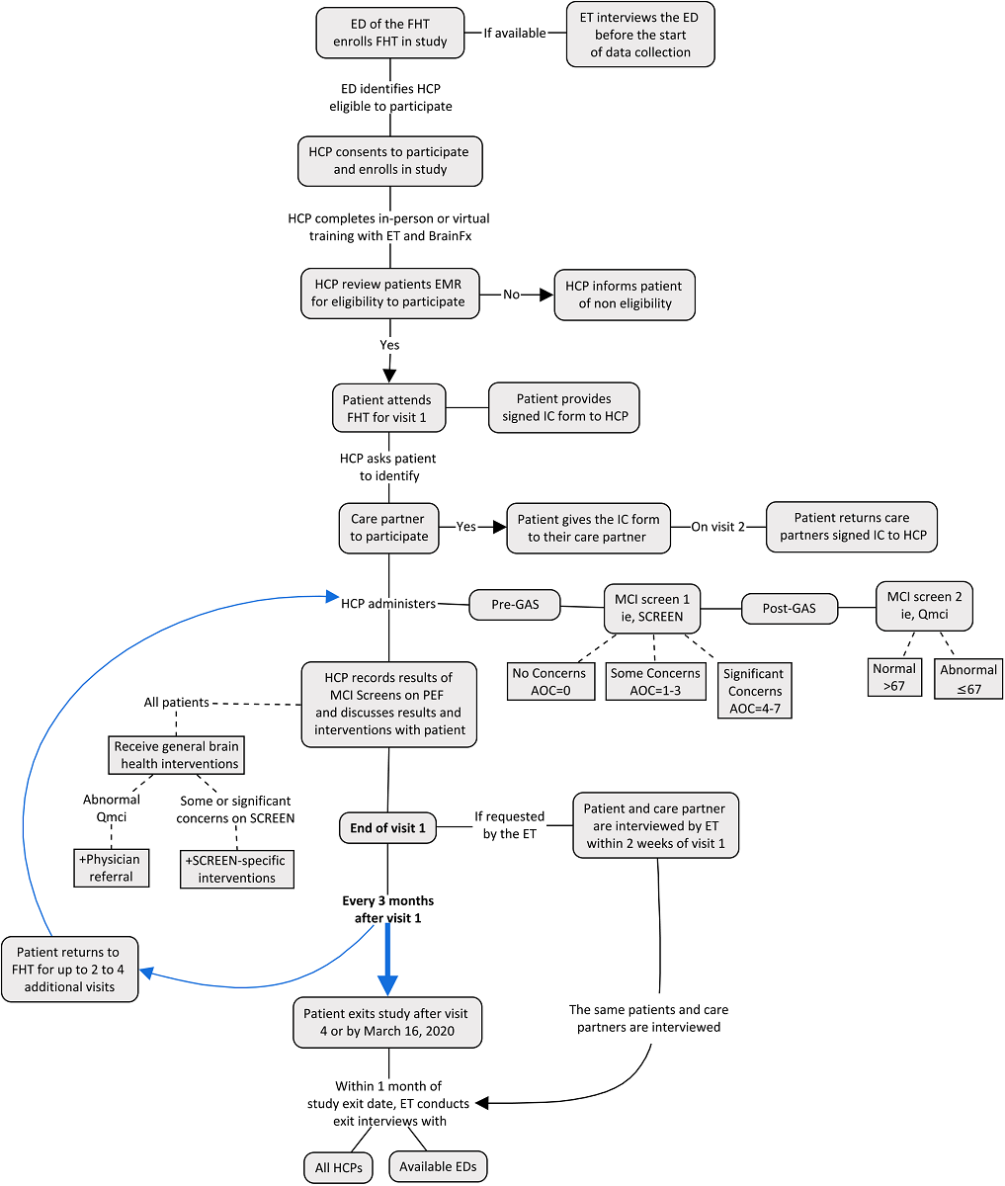
Study protocol data collection process. AOC: area of concern; ED: executive director; EMR: electronic medical record; ET: evaluation team; FHT: family health team; GAS: Geriatric Anxiety Scale; HCP: health care provider; IC: informed consent; MCI: mild cognitive impairment; PEF: patient encounter form; Qmci: Quick Mild Cognitive Impairment Screen.

The summary of the study protocol for the data collection process is included for reference in Figure 1.

#### Psychometric Evaluation

Data collection for the psychometric evaluation study will take place in person at the patients’ respective FHT. Standard operating procedures have been developed for the research study, which will be followed by the research team and HCP staff trained in the study protocol and administration of all data collection tools. A member of the research team will observe at least one in-clinic HCP data collection visit to confirm adherence to the protocol and training procedures.

To assess the reliability and perceived usefulness of the SCREEN, depending upon when a patient enters the study, they may repeat the screening protocol up to 4 times. Each visit is structured around the administration of 2 MCI screens; the *GAS-10* [81,82]; and a series of questions that measure the patient’s use of technology, change in their general health and well-being, or any interactions with the health care system related to MCI that occur in the 3 months since their last appointment. The HCP will be required to complete a patient encounter form at each appointment, which includes a summary of the patient’s MCI screen results, any referrals, and their responses to these questions.

The patients’ first appointment will take approximately 45-60 minutes (to account for onboarding), and all subsequent appointments (up to 4 screening test pairs, depending on the patient’s study entry date) will take approximately 45 minutes to complete. Rolling recruitment of patients will occur over an 18-month period and will end when a minimum of 2 and a maximum of 4 SCREEN-Qmci test pairs are completed every 12 weeks. In older adults, with no diagnosis of MCI or dementia, measures of cognitive abilities, including verbal fluency, attention, and intelligence [100], and measures of executive functioning [101] remain stable for anywhere from 4 to 8 weeks or from 1 to 5 years between test and retest (controlling for the effects of normal aging [100]). To date, there are no clear guidelines on the optimal time between tests [102,103]. Streiner [104] recommends longer periods to avoid recall bias. Furthermore, greater practice effects are experienced with shorter test-retest intervals [62]. The 3-month interval was therefore selected to minimize such confounds and is justified given the stability of the constructs under investigation. A randomization process will determine the order of screen administration at each visit. The Qmci will be administered using a pen and paper. HCPs will be provided with identical tablets, preloaded with the SCREEN app software.

The GAS-10 will be administered just before and immediately after the administration of the first MCI screen (eg, the SCREEN) and immediately followed by the administration of the second MCI screen (eg, the Qmci) at each appointment. After completing the 2 MCI screens, the HCP will manually calculate the results for the Qmci, log in to the BrainFx portal to retrieve the SCREEN report, and review both sets of results with the patient. The Qmci cut-off score for distinguishing MCI from normal cognition is ≤67/100 [88]. The SCREEN does not include scoring guidelines for a cut-off score but identifies whether a score on any of the 7 tasks is an *area of challenge*.

To determine the sensitivity and specificity of the SCREEN in comparison with the Qmci, the results of both screens will be classified in binary format as healthy or not healthy, where *healthy* denotes SCREEN=no areas of challenge in all 7 activities and Qmci≥67 and *unhealthy* denotes SCREEN=1 or more areas of challenge and Qmci≤67. Consistent with consensus guidelines for screening for cognitive impairment [37,49,50], the research protocol will also require HCPs to refer patients to their primary care physician for further evaluation if they receive an abnormal score on the Qmci or significant concerns on the SCREEN. The SCREEN does not have a standardized cut-off score that can be used to classify results as abnormal. Therefore, in consultation with and on the recommendation of the SCREEN developers, for the purpose of the research protocol, a set of cut-off scores was developed to classify SCREEN results as normal (zero areas of challenge), some concerns (≤3 areas of challenge), or significant concerns (≥4 areas of challenge). All HCPs in this study will be OTs who are trained to assess individuals’ abilities or disabilities through the use of standardized testing and functional observation. As a result, the study protocol includes a condition that referral decisions for physician oversight or further testing are the purview of the HCP and their clinical judgment. This became the proxy gold standard for a positive screen in this research study.

Following the review of the results at each appointment, the HCP will provide patients with a handout that summarizes recommendations for supporting cognitive health. If the SCREEN identifies any *areas of challenge,* the patient will also receive a handout developed by BrainFx containing tailored strategies to strengthen brain health and manage deficits in those areas.

#### Perceived Usefulness and Clinical Workflow Integration

**Figure 2:**
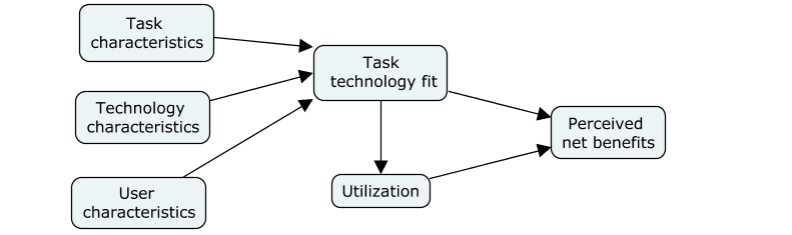
Task Technology Fit model.

Semistructured, in-depth interviews will be conducted, in person or by telephone, with HCPs, executive directors, and a subset of patients and their care partners throughout the study. Interviews will be audio-recorded and transcribed verbatim. Data collection will be guided by the *TTF theoretical model*, which emphasizes the importance of the fit between technologies and users’ tasks in creating a perceived net benefit to use [89,105] (Figure 2). Standard questions to capture demographics, attitudes, and experiences data will also be asked at the beginning of each interview.

HCPs will complete a TTF questionnaire (Textbox 2) as part of their entry or exit interviews with a member of the research team, which will be scheduled 3 months after they enter the study and again within 1 month after they exit the study.

**Task Technology Fit questionnaire. Task Technology Fit questionnaire response options are as follows: 1=entirely disagree, 2=mostly disagree, 3=somewhat disagree, 4=neither agree nor disagree, 5=somewhat agree, 6=mostly agree, and 7=entirely agree.**

**Technology enhancements**

- The quality of the information I receive from the BrainFx SCREEN and Report is enough to meet my clinical needs.
- The BrainFx SCREEN and Report provides me with the right data I need to better support patients and caregivers.
- I am able to quickly locate the results of the BrainFx SCREEN on a patient chart.
- The data elements on the BrainFx Report are easy to understand or it is easy to find out.

**User satisfaction**

- Technical support to access the BrainFx app was always available when I needed it.
- I can count on the BrainFx system to be “up and running” and available when I need it.
- The tablets were subject to unexpected or inconvenient downtimes, which makes it harder for me to do my work.
- The BrainFx app was subject to frequent problems.
- It was easy to learn the BrainFx system.

**Task characteristics**

- I frequently deal with nonroutine cases of older adults with cognitive issues.
- I frequently deal with routine cases of older adults with cognitive issues.
- Identifying cases of mild cognitive impairment usually takes more than one clinician.
- Sharing relevant and timely information with other care providers is important when diagnosing cognitive impairment.

**Utilization**

- The BrainFx system was convenient and easy to use.
- There was not enough training for me on how to administer the BrainFx SCREEN using the tablets.
- There was not enough training for me on how to use the BrainFx Report.
- BrainFx support took an interest in helping me to solve problems to avoid disruptions to my workflow.

**Perceived net benefits to use**

- My overall effectiveness in detecting mild cognitive impairment increased when I used the BrainFx SCREEN and Report.
- My ability to target interventions for individual patients and their needs was improved with the BrainFx SCREEN and Report.
- My ability to target interventions for individual caregivers and their needs was improved with the BrainFx SCREEN and Report.
- I waste less time interpreting test results and preparing interventions with BrainFx SCREEN and Report.
- I spend less time writing up charting cognitive test results with the BrainFx SCREEN and Report.
- The BrainFx SCREEN and Report provide better information to patients and their caregivers.
- The quality of my follow-up recommendations to patients and caregivers has improved with my use of the BrainFx SCREEN and Report.

### Statistical and Analytic Plan

Descriptive and inferential analyses will be conducted using SPSS Statistics for Windows, Version 26 (IBM Corp). Qualitative analysis will be conducted using NVivo version 12 (QSR International Pty Ltd).

#### Descriptive and Inferential Analysis

Descriptive data will be described using frequencies and percentiles and compared using the chi-square test or Fisher exact test as necessary. Continuous data will be analyzed for central tendency and variability; categorical data will be presented as proportions. Normality will be tested using the Shapiro-Wilk test, and nonparametric tests will be performed using the Mann-Whitney U test*.* Statistical significance will be considered at a *P* value of .05, with 90% CI provided where appropriate. We powered the exploratory analysis to validate the SCREEN using an estimated effect size of 12%, with the understanding that Canadian prevalence rates are not available [29], and determined that we needed at least 114 participants. For test-retest reliability, using 90% power and a 5% type 1 error rate, we will require a minimum of 58 test results.

MCI test outcome data will be coded into a binary format of *healthy* or *unhealthy* (where unhealthy indicates a positive result on the test, ie, 1 or more *areas of challenge* on the SCREEN results, or a score of ≤67/100 on the Qmci) to account for the difference in categorical versus continuous outcome variables. For this reason, sensitivity and specificity will be determined using cross-tabulation rather than using the area under the curve using receiver operating characteristic curves. A principal component analysis with varimax rotation will be used to better understand the derivation of components’ contribution to screen test outcomes and to explore the differences between a conventional MCI screen (Qmci) and one that is intended to be more ecologically valid by assessing functional impairment (SCREEN). Binary logistic regression will examine the effects of variables such as age, education, self-reported comfort with technology, anxiety before and after completing the MCI tests, and sleep levels on the results. The internal consistency of both the SCREEN and Qmci will be assessed using Cronbach α. Test-retest reliability, the ability of a measurement instrument to reproduce results on 2 or more occasions (ceteris paribus), will be assessed using an intraclass correlation coefficient [106].

#### Qualitative Analysis

To assess the perceived usefulness of adopting the tablet-based SCREEN in a real-world clinical setting, HCPs, FHT executive administrators, patients, and their care partners will be interviewed upon entry and exit from the study. All HCPs and patients will be interviewed twice, and care partners will be sampled until saturation [107]. Interviews will be audio-recorded and transcribed verbatim. Two members of the research team will analyze the transcripts using NVivo and a mix of inductive and deductive analytic techniques to identify themes and insights. Deductive insights will be drawn from sensitization to the TTF model to explore the impact of the software and hardware platform on the process of screening for MCI in a primary care setting.

## Results

This funded research was launched in January 2019, and enrollment was conducted in February 2020. Quantitative data collection was interrupted in March 2020 because of the COVID-19 pandemic and the shutting down of all nonessential in-person clinical visits; qualitative data collection was concluded in July 2020. The results are forthcoming.

## Discussion

### Summary

This research study will assess the psychometric properties and perceived usefulness of a novel tablet-based tool to screen for MCI in adults 55 years and older in the primary care setting. A fundamental objective of a screening test is to reduce morbidity or mortality in an at-risk population through early detection and treatment [108], with the anticipated benefit outweighing potential harm [58]. However, a rapid screening test for MCI might also assist time-strapped, cost-sensitive primary care physicians [109] in determining whether referral for a definitive battery of more expensive tests is warranted [110]. Screening for MCI and dementia is usually conducted through informant reports of functional impairment [14] and patient performance outcomes on tests with high sensitivity [58], with no consensus or guidelines on which are most effective [63,109-112]. Many of these tests are inappropriate for use in primary care because they are time-consuming to administer, insufficiently sensitive, ecologically invalid, or impacted by education or cultural bias. In addition, advances in the field of neurobiology are changing our understanding of MCI and testing, for instance, tests of object discrimination and familiarity may be better suited to detect mild dysfunction from MCI as they rely on intact functioning of the perirhinal cortex of the hippocampus, which is impaired in the earliest stages of MCI [25]. Identifying affordable, psychometrically tested screening tests for MCI that conform to clinical workflows and are easy to consistently administer and complete may initiate treatment if appropriate, help normalize and destigmatize cognitive testing for older adults, expedite referral, allow early access to programs and services that can support aging in place or delay institutionalization, and improve the psychosocial well-being of patients and their care partners by increasing access to information and resources that aid with future planning and decision making [113].

### Limitations

The assessment of this novel screen for MCI will be executed within the constraints of limited financial resources. As such, it will methodologically constrain our use of a presumed *gold standard* test for MCI with the participant population, which in many studies involves a neuropsychological evaluation. In fact, the presence or absence of a gold standard MCI screening test is the subject of some debate [112]. The absence of an established gold standard has resulted in the use of proxies, such as a validated, sensitive test for the same condition [107,108].

Although patient participation in the study is voluntary (there is no randomization or selection for particular traits), we anticipate that those with concerns about their cognition and those more comfortable with the use of tablet technology may be more likely to self-select into the study. Our data collection and statistical analysis will account for both the potentialities.

## Abbreviations

ADL: activities of daily living
EMR: electronic medical record
FHT: family health team
GAS: Geriatric Anxiety Scale
HCP: health care provider
IADL: instrumental activities of daily living
LBB: Living Brain Bank
MCI: mild cognitive impairment
MMSE: Mini-Mental State Exam
MoCA 8.1: Montreal Cognitive Assessment 8.1
Qmci: Quick Mild Cognitive Impairment
TTF: Task Technology Fit
ZBI: Zarit Burden Interview
